# Intestinal Auscultation

**Published:** 1849-10

**Authors:** 


*Intestinal Auscultation.*—Prof. Charles Hooker, of the Medical Institution of Yale College, Conn., has published some observations on the subject of Intestinal Auscultation, which are curious, if not important. Every effort to increase our means of diagnosis is deserving attention, and, possibly, in this new field of inquiry may be developed valuable signs for the discrimination of intestinal affections. Prof. H. has published his observations in a pamphlet form, reproducing communications which first appeared in the Boston Med. and Surg. Journal. We quote his remarks on the auscultatory phenomena present in Asiatic cholera, and colic:—

"In the Asiatic Cholera, which prevailed in New Haven in 1832, this application of auscultation was attended with interesting results, which were noticed in an account of the cases which came under my observation, published in the Boston Medical and Surgical for July, 1833. Writers generally noticed the loud borborygmi, audible at a distance from the patient, which occurred in that disease; and to the ear applied over the abdomen the sounds were so peculiar—at least so different from what I have observed in other diseases—that they seemed distinctly characteristic of that disease. These sounds manifested a rapid commotion of the whole intestinal canal, and might be compared to those produced by shaking together several flasks of various sizes partly filled with water. Frequently the sounds appeared to indicate that the rapid peristaltic motions were suddenly arrested and reversed by an anti-peristaltic action, which occurrence immediately preceded a paroxysm of vomiting. The large quantity of serum effused into the intestines, causing an extreme fluidity of their contents, with the rapid and irregular peristaltic motions, would sufficiently account for this unusual variety of sounds.*

The effects of various remedies upon the intestinal action, as indicated by the sounds, were carefully observed. Practitioners were generally disappointed, in that disease, to find the frequent vomiting and purging not checked by the administration of stimulants and astringents; and the sounds manifestly indicated that the common effect of these remedies was decidedly to increase the intestinal commotion. Such was the manifest effect of opium, unless given in doses so large as to produce alarming prostration. On the other hand, frequent small doses of camphor, with a free administration of ice, appeared to have a soothing operation in moderating the rapid and irregular intestinal action. The comparative effects of large and small doses of calomel were strikingly interesting. Frequent small doses did not seem to diminish, but at least temporarily to increase, the disordered peristaltic and anti-peristaltic motions; while a single drachm dose almost invariably caused a total suspension of these motions. Calomel, in very large doses, thus seemed to be the appropriate remedy for the disease. It appeared to overpower the diseased intestinal action, arrested

* It remains to be shown, whether these sounds are constant diagnostic signs of this disease, or whether, as I have observed in dysentery and other diseases, the varying epidemic type, in different seasons, will produce in cholera a variation of morbid intestinal action, with a variety of sounds.

the vomiting and purging, and caused a total suspension of all intestinal motion, during which no sound was audible. An interval of perfect intestinal silence and repose now continued, ordinarily from eight to twelve hours, after which a natural peristaltic murmur indicated a gradual return of healthy action, which was in time succeeded by the grass-green evacuations, commonly regarded as evidences of a favorable crisis of the disease. Thus the large doses of calomel, instead of exhausting the system by an excessive cathartic operation, actually obviated exhaustion by arresting the profuse serous evacuations attending the disease.

Ordinarily the danger was considered as overcome, when the disordered intestinal action was suspended, and the stage of repose produced; and in this town few cases terminated fatally, when the practice was adopted of effecting this result by the large doses of calomel, before the system had been extremely exhausted by evacuations. In one case, however, that of a little girl, 10 years of age, who, without any premonitory symptoms, was most violently attacked with vomiting, purging and spasms, this treatment had the ordinary effect of promptly arresting the intestinal motions; but the system did not re-act, the pulse failed and became imperceptible within an hour from the attack.

*Cholera Morbus* is usually attended with intestinal sounds, which indicate a succession of quick and irregular peristaltic and anti-peristaltic motions. In some cases these motions continue until the contractile power of the intestines seems nearly exhausted, when a feeble, but more regular, peristaltic murmur indicates a gradual return of healthy action. The violent symptoms are not succeeded, as in Asiatic cholera, by a long interval of total inaction of the intestines; and the sounds are very different from those heard in that disease.

There is, however, a great diversity in cases commonly termed cholera morbus. Some cases commence with a violent diarrhœa, on the cessation of which occurs an obstnate vomiting, dnring which, as in colic, no intestinal sounds aie heard, except those produced by anti-peristaltic action. Other cases commence with vomiting, without any downward motions, until at leugth the action is reversed, and the disease terminates with diarrhœa.

*Colic* is a disease which is variously divided by writers into several species. One of these, termed c. rachialgia, c. pinctonum, &c., produced by the poison of lead, has characteristics certainly sufficient to give it a specific distinction; but the other divisions, I think, have reference to various exciting causes, or attendant circumstances, rather than to any proper specific characters. In the various forms of this disease auscultation affords results, which I regard as highly interesting, and of much practical value, and which may throw some light on the pathology of the disease.

*Common Colic* is characterized by "griping pain in the bowels, chiefly about the navel, with vomiting and costiveness." The exciting causes are various, as irritating indigested food, biliary derangement, habitual costiveness, hardened fæces, flatus, worms, exposure to cold, and—what I consider as much the most common cause—rheumatism affecting the intestines. With these various exciting causes, the general characters of the disease are similar; the severe griping pain, obstinate constipation and vomiting, constituting the prominent symptoms.

There is, however, an *incipient, forming, or latent stage,* which with strict observation I think may always be noticed, preceding the pain and other violent symptoms. The symptoms of this stage somewhat resemble those which precede the cold stage of intermittent fever. There is a general languor and inaptitude, often a degree of moroseness or peevishnesss, and commonly a slight chilliness. The sensations in the abdomen are variously described by patients, as a numb, dead, heavy, or cold feeling. Many speak of a sensation as of a cold weight, felt mostly between the region of the stomach and umbilicus. The physician is rarely consulted during this stage; and the symptoms are so slight, that ordinarily they are not particularly noticed by patients unaccustomed to attacks of the disease; while persons subject to frequent attacks learn to notice these sensations as the invariable precursors of the more violent symptoms. In this stage, which continues in different cases from half an hour to several hours, auscultation discovers a perfect stillness within the abdominal cavity. Sometimes there is an occasional rumbling in the course of the large intestines; and, with a desire to relieve the unpleasant sensations, the patient by a voluntary straining effort produces an evacuation of fæces with a quantity of flatus. There is, however, no indication of the slightest motion in the small intestines. This forming or latent stage of colic, which is commonly overlooked both by patients and physicians, is deserving of particular attention; because during this stage the peristaltic action is easily restored, and the violent symptoms thus prevented. In many cases this may be effected simply by the application of heat to the surface, especially to the extremities. Friction to the abdomen, with a sort of kneading process, contributes also to this effect. Often a free draught of hot coffee, or of some aromatic infusion, is sufficient; in other cases, a small dose of rhubarb, or other mild cathartic, with some aromatic, is required. A few drops of cajeput oil will commonly promptly restore the peristaltic action. My usual remedy for this purpose is camphor, in frequent small doses; and I have instructed many persons to ward off habitual attacks of colic, bycarrying constantly in the pocket a small piece of camphor, to be gradually dissolved in the mouth, and swallowed with the saliva, whenever these premonitory symptoms occur. This remedy is often more effectual, in exciting peristaltic action in such cases, than a brisk cathartic.

This forming stage, unless the peristaltic action is soon restored, is succeeded by the violent symptoms of the disease. With occasional short remissions, the pain becomes severe; the abdominal muscles are rigidly contracted, producing a knotted appearance of the surface, and there is occasional nausea and vomiting. The patient groans, and throws himself into various positions, with the vain hope of relieving his distress. In this, as well as in the forming stage of colic, the ear applied to the abdomen discovers no evidence of peristaltic action, but on the contrary a perfect stillness within the abdominal cavity.

This *cessation of peristaltic action*, I may confidently assert, is a chief *essential character of colic;* the motion being suspended before the occurrence of the violent symptoms, and not recurring until the disease is about to yield. Sometimes, during the violent contortions of the body, a momentary sound is heard, indicating a slight intestinal motion, which seems to be produced by the mechanical pressure of the abdominal parietes, rather than by a peristaltic action. Occasionally, too, there are sounds produced by anti-peristaltic motions, which motions either terminate at the stomach causing simply nausea, or extend into the stomach so as to excite vomiting. By these circumstances, and by the variety of sounds, anti-peristaltic motions can commonly be distinguished from a regular peristaltic action. This distinction is important, for as a cessation of peristaltic action is a main essential character of colic, so a return of this action indicates a favorable crisis of the disease. The sounds produced by anti-peristaltic motions are only occasional and transient, proceeding commonly from a limited portion of the intestinal canal; and they are usually succeeded, as before stated, by nausea or vomiting. Those attending a regular peristaltic action are produced throughout the whole course of the intestines, constituting an almost incessant rumbling, heard distinctly at one moment directly under the ear, then gradually receding until it seems like a distant echo, and again returning in the course of the convolutions of the intestines. There is thus a union of near and distant sounds, indicating a general action throughout the intestinal canal. When this description of sounds is heard in colic, the patient may be considered as safe, even if the pain continues severe; on the contrary, a complete subsidence of the pain and other violent symptoms, unless attended by a return of healthy peristaltic murmur, affords no favorable indication, in any stage of the disease, and in an advanced stage, when the strength is exhausted by protracted suffering, it indicates extreme danger—a loss of the sensibility and excitabilily of the intestine, and a failing of the powers of life.

Commonly, a return of peristaltic motion is followed, almost immediately, with a relief of pain and other severe symptoms; but in protracted cases, when the bowels have become inflamed, and the soreness such that the least external pressure cannot be tolerated, this return of peristaltic motion causes a decided increase of pain. This circumstance is similar to what is often observed during the resolution of pneumonia, when a return of respiration to a portion of inflamed lung, which has previously been impermeable to air, produces the keenest pain. In such cases auscultation informs us that all is well, when the sensations of the patient would indicate an aggravation of the disease. The signs thus furnished, in colic and other diseases, will often direct the withholding of medication, when it is no longer required, and when its continuance might sometimes be injurious. Frequently they have enabled me to assure patients that the cause of difficulty was removed, and that my services were no longer required, some hours before the general symptoms showed signs of any mitigation."

